# Effect of water salinity on immature performance and lifespan of adult Asian tiger mosquito

**DOI:** 10.1186/s13071-023-06069-5

**Published:** 2024-01-18

**Authors:** Laura Blanco-Sierra, Eleni C. Savvidou, Evangelia D. Mpakovasili, Charalampos S. Ioannou, Frederic Bartumeus, Nikos T. Papadopoulos

**Affiliations:** 1 Centre d’Estudis Avançats de Blanes (CEAB-CSIC), Carrer d’Accés Cala Sant Francesc, 17300 Blanes, Girona Spain; 2 Laboratory of Entomology and Agricultural Zoology, Department of Agriculture, Crop Production and Rural Environment, University of Thessaly, Phytokou Str, 38446 Nea Ionia, Magnesia Greece; 3 ICREA, Institució Catalana de Recerca i Estudis Avançats, Passeig de Lluís Companys, 23, 08010 Barcelona, Barcelona Spain; 4 CREAF, Ecological and Forestry Applications Research Centre, Campus de Bellaterra (UAB), 08193 Barcelona, Barcelona Spain

**Keywords:** *Aedes*, Dispersal, Mosquito, Salinity, Survival

## Abstract

**Background:**

*Aedes albopictus* (Skuse, 1894) is a vector for pathogens like dengue, chikungunya, and Zika viruses. Its adaptive capacity enables reproduction in temperate climates and development mainly in artificial containers with fresh water in urbanized areas. Nevertheless, breeding in coastal areas may also occur along with its aggressive invasiveness. Global warming and the consequent rise in sea levels will increase saline (> 30 ppt) or brackish (0.5–30 ppt salt) water in coastal regions. To address whether *Ae. albopictus* can breed in brackish water, we initiated the current study that analyses the survival of immature stages at different salinity concentrations and explores whether carryover effects occur in the resulting adults. This possible adaptation is important when considering the potential for development in new habitats and expansion of one of the world’s most invasive species.

**Methods:**

We investigated the influence of salinity on the survival of *Ae. albopictus* larvae and adults under laboratory-controlled conditions. First instar larvae were exposed to different salinity concentrations (0 to 30 ppt) and their development time, pupation, adult emergence, and overall survival were monitored daily. We used Kaplan-Meier and Cox regression models to analyze the survival rates at different salinity levels. Furthermore, life tables were constructed under each salinity concentration.

**Results:**

Increasing salt concentrations significantly increased the mortality risk during immature development, while no significant effect was observed on adult mortality risk. A comparison between distilled and bottled water revealed a notable increase in overall mortality risk for individuals developing in distilled water. However, no significant effects were found when analyzing survival from the first larval stage to adult emergence and adult lifespan. The life expectancy of immature stages decreased with increasing salt concentrations, although salinity concentration did not significantly impact adult life expectancy.

**Conclusions:**

Our findings suggest that *Ae. albopictus*, previously considered freshwater species, can successfully develop and survive in brackish waters, even in the absence of characteristic structures found in euryhaline species. These adaptations may enable *Ae. albopictus* to establish new breeding sites and colonize unexplored territories. Knowledge of these physiological adaptations of *Ae. albopictus* to salinity should be pursued to increase the range of control of the species, and to make more accurate predictions of its dispersal and vectoring ability.

**Graphical Abstract:**

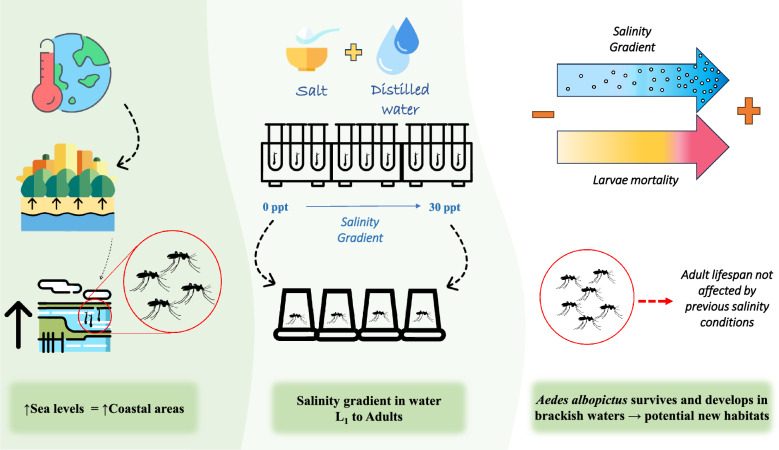

**Supplementary Information:**

The online version contains supplementary material available at 10.1186/s13071-023-06069-5.

## Background

*Aedes albopictus* [1], commonly known as the Asian tiger mosquito, is a species of great importance for public health since it is a vector for various pathogens, including chikungunya, Zika, and dengue viruses [2, 3], as well as filarial worms such as *Dirofilaria immitis* and *Dirofilaria repens* [4]. Originating from Southeast Asia, during the latter half of the twentieth century, *Ae. albopictus* expanded its geographical range to numerous countries worldwide [5]. The Asian tiger mosquito lays desiccation-resistant eggs, and its immature stages develop in both natural and artificial freshwater collections, such as human-made containers [6]. Both of the above traits enable survival during transportation (e.g. trading of used tires and lucky bamboos) over long distances, including regional and intercontinental travel [7, 8]. The rate of its global spread has strongly correlated with increased globalization in trading in recent decades [9]. *Aedes albopictus* was first reported in Albania in 1979 [10] and has since dispersed and establish itself in over 30 European countries [11]. It is associated with major disease outbreaks in Europe (e.g. dengue and chikungunya in Croatia, France, Spain, and Italy [12, 13]). Coastal areas with Mediterranean climates are current hot-spots for its European establishment. However, its adaptability to cooler environments, winter survival as diapausing eggs, and projected temperature rise from global warming may further promote its expansion [14, 15]. Numerous studies have examined the impact of climate change on the epidemiology of mosquito-borne diseases, focusing mainly on the influence of temperature, rainfall, humidity, wind, and particle pollution on mosquito vectors [16, 17, 18, 19]. Among secondary parameters are the global distribution and characteristics of plants and animals, the frequency and severity of extreme weather events, and global rise in sea levels [19]. Extreme events like floods and droughts are likely to become more frequent, and heavy rainfalls may provide standing water surface required for egg-laying and larval development in some mosquito species, such as *Ae. albopictus*. In addition, projected rise in sea levels will likely lead to higher occurrences of saline and brackish water environments in coastal regions, including estuaries, marshes, and other areas[20]. Estuarine regions are expected to experience increased salinity levels, accompanied by greater tidal flows into rivers [21, 22]. These changes have the potential to reshape coastal ecosystems and influence the distribution of mosquito populations and associated diseases. In fact, previous studies have shown that a rise in sea levels could increase the prevalence of many vectors of pathogens in coastal zones [23].

Mosquito larvae can inhabit various types of water, including fresh, brackish, and saline waters [24], categorized by their salt content (< 0.5 parts per thousand [ppt], 0.5–30 ppt, and > 30 ppt, respectively) [25]. Osmoregulatory mechanisms differ between larvae developed in fresh water and those found in saline water [26]. Freshwater species are obligate hyperregulators, while euryhaline species (able to develop in saline waters) are hyperregulators in diluted environments and hyporegulators in saline environments [27]. Euryhaline mosquitoes possess a rectum comprising two segments: an anterior segment functioning as the rectum in freshwater forms, and a posterior segment involved in salt secretion. This adaptation enables these species to survive in saline waters [28, 29].

 Studies have explored the saline tolerance of larvae belonging to the genera *Culex* and *Culiseta* demonstrating high levels of organic compounds in larvae haemolymph resulting from an osmoregulatory response stemming from a distinct evolutionary trajectory [27]. Among the *Aedes* genus, certain species such as *Aedes detritus* and *Aedes dorsalis* exhibit resistance to saline water [26] and have likely adapted to lay eggs and undergo development in such breeding sites. Another prominent example is the so-called *Aedes mariae* complex, which inhabits rock pools along the coasts of the Mediterranean basin [30]. These rock pools along the sea coast are considered extreme habitats because their salt concentration can range from almost fresh water to 250% seawater [31]. It has also been observed that immature development of the freshwater mosquitoes *Ae. aegypti* and *Ae. albopictus* can occur in brackish water collections in unused wells and discarded artificial containers [23, 32]. The ability of the latter freshwater species to tolerate salinity in its immature development has been further studied [33, 34]. In the genus *Anopheles*, species such as *Anopheles stephensi* evidence the ability to breed in brackish water [35].

The aim of the current study was to explore the response of immatures of *Ae. albopictus* to water salinity and the potential effects on adult life history traits. Specifically, we tested the survival and developmental time of the different larvae instars breeding in water of different salinity levels, as well as the lifespan of the emerging adults. Hence, we addressed both direct and indirect effects of water salinity on critical aspects of the life cycle of *Ae. albopictus*. Our work provides data to support future predictive studies of its possible prevalence in brackish water bodies in coastal areas as a salinity-tolerant mosquito.

## Methods

### Laboratory conditions and colony rearing

**Fig. 1:**
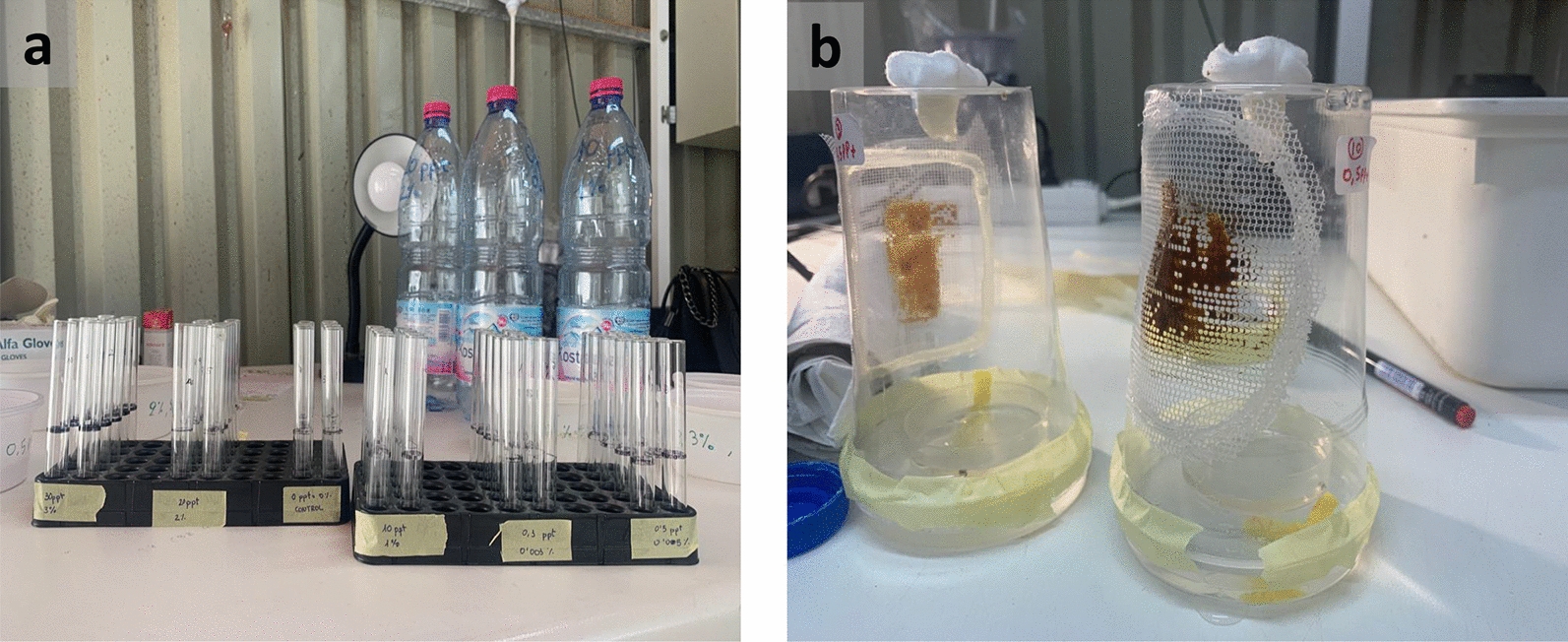
**a** Tubes containing single individuals from L1 to L4 stages. **b** Individual cages for pupal stages. Once the adults emerged, they stayed in the same cages, identified by their number and salt concentration

The experiments were conducted in the laboratory of Entomology and Agricultural Zoology at the University of Thessaly, Greece, from April to October 2022. We utilized walk-in chambers set at a temperature of 25 ± 2 $^{\circ }$C, relative humidity of 65 ± 5%, and a light cycle of L14:D10. The light intensity was gradually adjusted to simulate sunrise and sunset, using fluorescent cold-light lamps with a range of 800 to 1000 Lux. For the experiments, we used a laboratory-adapted colony of *Ae. albopictus* established in 2017 from eggs collected in the vicinity of Volos and Larisa city (Thessaly, Greece). The mosquito population has been reared for several generations to establish a uniform genetic background. For more details regarding colony origin and adaptation to laboratory conditions, see Ioannou et al. [36]. To establish different salinity concentrations, we based the solutions on distilled water and added varying amounts of NaCl: 0.2, 0.5, 1, 2, 5, 10, 12, 15, 20, and 30 ppt. Additionally, we established two controls with no addition of salt, using distilled water and bottled water (see Additional file 1: Table S1), respectively. To investigate the survival of *Ae. albopictus* larvae under different salt concentrations, we individually placed first-instar larvae (L_1_) into glass tubes (10 cm height, 1 cm diameter) that we had previously cleaned with soap, rinsed with distilled water, and dried in an oven at 150$^{\circ }$C for 2 h. Each tube contained 3 ml of the respective water solution and 0.1 g of milled cat food (Purina, Friskies). For each water solution treatment, we used 50 larvae, and the experiment was repeated twice. As in other demographic studies, each individual was considered a single replicate, as we focused on the survival of each individual at each salt concentration [37, 38, 39]. We monitored the tubes daily at the same hour (±1 h) and recorded mortality, developmental duration, and larval instar. After pupation, we transferred the pupae individually to small petri dishes (*r* = 5 cm) filled with water of corresponding salinity. Each petri dish was placed inside a small individual cage created by fitting a plastic cup (400 ml) into a plastic petri dish lid with a small hole, ensuring that emerging adults were already in their individual cages. We covered the cage openings with nylon mesh to allow for ventilation (Fig. 1). We provided water with a small wick cloth into the hole of each petri dish in the cage, and we supplied organic molasses as the adult mosquitoes’ food source through the mesh. We recorded the mortality of emerging adults daily and renewed the water and food resources as needed.

### Statistical analysis

We conducted binomial logistic regression to evaluate the effect of increasing salinity on the proportion of L$_1$ reaching (i) pupal stage and (ii) adult stage. To examine the proportion of L$_1$ reaching pupal and adult stages in the conditions without the addition of salt (i.e., distilled and bottled groups), we employed a Chi-square test and performed a residual analysis for pairwise comparisons. Since developmental durations did not conform to a normal distribution, we employed the Kruskal–Wallis tests to identify any significant differences in the durations for the stages from L$_1$ to pupal development, pupal to adult development, and adult lifespan in the different salinity conditions, followed by the Dunn test for pairwise comparisons ($\alpha$ = 0.05).

The Kaplan-Meier method was used to estimate survival functions for each salinity concentration, considering the immature (L$_1$ to adult emergence) and adult lifespan alone [40]. Censored observations (*n* = 105) included both adult mosquitoes that escaped and individuals who died in an artificial environment within the tubes (e.g. human-mediated causes). We performed pairwise comparisons on survival among the different salinity treatments using the Mantel–Cox log-rank test. For the analysis of survival of immatures, lifespan of adults, and total lifespan (L$_1$ to adult death), Cox regression hazard models were used to infer effects of salinity and sex on the length of the whole life course and lifespan of adults. Life tables were estimated for each salinity treatment following the methodology of Carey and Roach [41] (Additional file 1: Table S2). The analyses were performed using the *survival* analysis package [42] and *survminer* package [43] in RStudio 4.2.0 software [44]. Life tables were calculated using Microsoft Excel (version 2019).

## Results

**Fig. 2:**
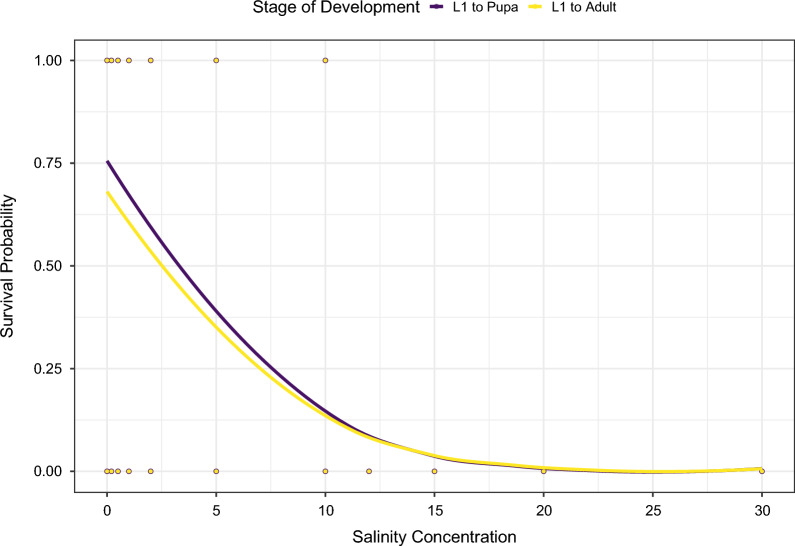
Regression curves for the analysis of the effects of salinity on the survival from L1 to pupa (purple) and L1 to adult (yellow). For more information, see Table 1

Binomial logistic regression analysis revealed that increasing salinity conditions had a significant and negative effect on the proportion of individuals surviving to pupal stage and the proportion of emerging adults (Table 1). The survival rates of immatures declined rapidly in response to salinity concentration from approximately 75% in distilled water to less that 25% at 8 ppt salt, while survival ceased in salinity levels higher than 12 ppt (Fig. 2). The similarity in survival probability between L$_1$ to pupae and L$_1$ to adult demonstrates low effects of salinity on pupa. The proportion of L$_1$ individuals successfully pupating and yielding adults was higher in the bottled water condition than in the distilled water condition ($\chi^2$= 27.31, 36.13; *df*= 1, *P* < 0.0001 for individuals surviving until pupa and adult emergence, respectively). Additional file 2: Fig. S1 provides event history diagrams depicting the progression of mortality and development of individuals under different salinity conditions.

**Table 1:** Results of the binary logistic regression on the effect of increasing salinity to survival rates from L1 to emergence of adults and overall survival

Effect	b	Exp(b)	SE	*z*-value	Pr($>|z|$)
*L1 to Adult emergence*					
Intercept	0.981	2.668	0.101	9.658	< 0.0001
Salinity	$-$0.280	0.755	0.020	$-$13.495	< 0.0001
*Overall survival*					
Intercept	0.683	1.981	0.097	7.018	< 0.0001
Salinity	$-$0.258	0.772	0.020	$-$12.538	< 0.0001

Distilled condition used as 0 and bottled water excluded from the analysis

Table 2 illustrates the duration of each developmental stage under various salinity conditions. Salinity concentrations exceeding 12 ppt resulted in a 1-day duration for the L$_1$ stage due to high mortality within the first 24 h. However, salinity concentrations below 12 ppt exhibited similar average durations for each developmental stage, although some significant differences were observed among certain salinity conditions. For instance, individuals developed at 10 ppt demonstrated a shorter adult lifespan than those grown at lower salt concentrations (Table 2).

**Table 2:** Effects of water salinity on immature developmental duration in days and the lifespan of the resulting adults

Salinity	L$_1$ to pupation	Pupa to adult	Adult lifespan
0.2	10.42 ± 1.31$^{a,b}$ (8–18)	3.05 ± 0.77$^{a}$ (1–6)	45.09 ± 25.16$^{a,b}$ (2–102)
0.5	10.76 ± 1.00$^{a,b,c}$ (8–14)	2.90 ± 0.60$^{a,b}$ (0–5)	29.19 ± 26.07$^{c}$ (1–98)
1	10.71 ± 0.91$^{a,b,c}$ (8–14)	2.82 ± 0.51$^{a,b,c}$ (0–4)	48.19 ± 27.56$^{a,b}$ (1–94)
2	10.81 ± 1.23$^{a,b,c}$ (8–15)	2.97 ± 0.72$^{a}$ (1–5)	45.38 ± 31.45$^{a,b,c}$ (1–103)
5	11.08 ± 1.93$^{ac}$ (8–22)	2.89 ± 0.64$^{a,b,c}$ (1–4)	49.20 ± 27.53$^{a,b}$ (1–98)
10	12.07 ± 2.29$^{c}$ (8–18)	2.88 ± 0.65$^{a,b,c}$ (0–4)	29.5 ± 27.36$^{b,c}$ (1–94)
12	–	–	–
15	–	–	–
20	–	–	–
30	–	–	–
Bottled	9.75 ± 1.56$^{d}$ (6–21)	2.58 ± 0.59$^{c}$ (0–4)	52.51 ± 24.58$^{a}$ (4–100)
Distilled	10.17 ± 1.28$^{b,d}$ (8–13)	2.5 ± 0.85$^{b,c}$ (0–4)	42.13 ± 29.38$^{a,b,c}$ (1–97)

Within each column values followed by different superscript letter are significantly different (Dunn test following a significant Kruskal–Wallis non-parametric analysis of variance [ANOVA], *P* < 0.05). Min–max values defining range are given in parentheses

**Fig. 3:**
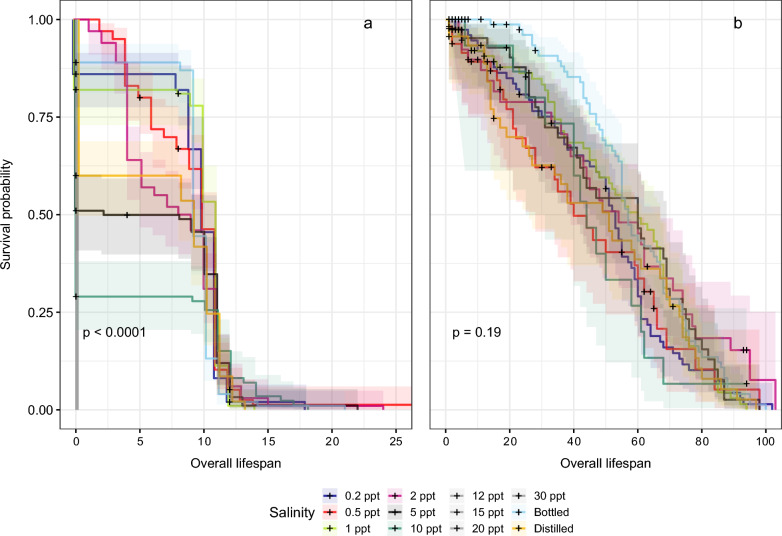
Kaplan–Meier survival curves of **a** survival from L$_1$ until adult emergence and **b** the adult lifespan for individuals across all the salinity experimental conditions. Individuals from 12, 15, 20, and 30 ppt died in the first 24 h as L$_1$, none of them reaching adult stages

The age-specific cumulative survival curves for the different salinity condition groups and the groups with no addition of water, both for L$_1$ until adult emergence (a) and for adult lifespan (b), are given in Fig. 3. In the immature cumulative survival curves, we observed higher survival rates in the bottled water than in the other salinity conditions and distilled water (log-rank test, *P* < 0.0001) (Fig. 3a). Additionally, in individuals developing at 0.2 ppt, 0.5 ppt, 1 ppt, 2 ppt, 5 ppt, and 10 ppt, we obtained a slight decrease in L$_1$ to adult emergence survival rates with increasing salt concentration. Generally, higher salinity concentrations corresponded to lower survival rates at the end of immature development, and pairwise comparisons (Additional file 1: Table S3) revealed significant differences among groups (*P* < 0.0001). However, we found lower survival rates at the 0.5 ppt than the 1 ppt condition for immature stages (Fig. 3a). Salinity concentrations above 12 ppt resulted in no survival of immature stages, and all individuals died within the first 24 h at concentrations up to 15 ppt. The survival of adult mosquitoes was independent of the salt condition in which they were reared as larvae (log-rank test, *P* = 0.19), exceeding all conditions at a lifespan of 80 days (Fig. 3b). Similarly, no significant differences were found across salt conditions when comparing the survival of adults segregated by sex (log-rank test, *P* = 0.29) (Additional file 2: Fig. S2).

We performed Cox regression models to analyse different datasets, taking the overall survival, L$_1$ to adult emergence, and adult lifespan (Additional file 1: Tables S4 and S5). All models were performed considering salinity as a continuous variable and distilled condition set as 0 ppt. Bottled condition was excluded from the analysis, as it contained some ions and salts in its composition (Additional file 1: Table S1). Results revealed increasing salinity as a significant predictor of the risk of death at overall survival (HR = 1.15; 95% CI 1.13$-$1.16; *P* < 0.0001), as well as from L$_1$ to adult emergence (HR = 1.130; 95% CI 1.12$-$1.14; *P* < 0.0001). Similar analysis revealed that the lifespan of males was slightly shorter than that of females (i.e. the risk of death was higher in males than in females) (HR = 1.31; 95% CI 1.04$-$1.66; *P* < 0.05). However, the salinity of water during immature development was not a significant predictor of adult longevity (HR = 0.99; 95% CI 0.94$-$1.04; *P* = 0.661). Considering only the conditions with no salt addition on the overall survival, we found that distilled water was associated with a higher risk of death compared with bottled water (HR = 1.91; 95% CI 1.42$-$2.58; *P* < 0.0001). However, the comparison between distilled and bottled water revealed no significant risk of death when analysing separately the survival from L$_1$ to adult emergence (HR = 1.06; 95% CI 0.80$-$1.41; *P* = 0.672) and adult lifespan (HR = 1.29; 95% CI 0.88$-$1.89; *P* = 0.193). There were also no significant differences in the risk of adult death by sex in these conditions (HR = 0.829; 95% CI 0.56$-$1.21; *P* = 0.338).

Life expectancy of individuals was calculated by constructing life tables for each salinity condition, from L$_1$ to adult death and the adult period, segregated by sex. The lowest life expectancy was observed at salt concentrations of up to 12 ppt (< 1 day) regarding the complete life cycle, while in adults, the lowest life expectancy was found at 10 ppt in females (43.21 days) and 2 ppt in males (38.88 days) (Table 3). Comparatively, individuals from the distilled condition had approximately half the life expectancy at age 0 as those from the bottled water condition when considering the complete cycle (31.01 days vs 59.98 days, respectively). The effect of exposure to different salinity concentrations during their immature period was not that clear when individuals reached adult stage. In the adult lifespan, we still observed higher life expectancies at age 0 from the bottled condition relative to distilled water, for both males (61.45 vs 46.20 days) and females (54.36 vs 45.22 days), respectively (Table 3).

**Table 3:** Key statistics extracted from the life tables for the different salinity conditions, including the life expectancy (in days) both at age 0 for the complete life course of individuals and for the adult period, segregated by sex

Salinity condition	Number of mosquitoes in each condition	Number of mosquitoes reaching adult stage	E$_x$ at age 0 (L$_1$ to adult death)	Maximum age (in days) reached (L$_1$ to adult)	E$_x$ at age 0 in females (adult lifespan)	E$_x$ at age 0 in males (adult lifespan)
Bottled	100	85	59.98	106	54.36	61.45
Distilled	100	46	31.01	106	45.22	46.20
0.2 ppt	100	76	48.04	113	45.57	50.21
0.5 ppt	100	54	35.41	109	51.54	39.95
1 ppt	100	76	51.70	105	55.66	48.50
2 ppt	100	41	29.35	114	71.33	38.88
5 ppt	100	40	31.73	109	51.92	54.19
10 ppt	100	24	16.87	105	43.21	47.50
12 ppt	100	0	$<1$	3	–	–
15 ppt	50	0	$<1$	$<1$	–	–
20 ppt	50	0	$<1$	$<1$	–	–
30 ppt	50	0	$<1$	$<1$	–	–

## Discussion

Despite being typically associated with fresh water, this study provides clear evidence of *Ae. albopictus*’ ability to survive and develop in brackish water. This research holds significance in expanding our knowledge about the species’ potential to thrive in novel breeding environments. *Aedes albopictus* has already demonstrated its ability to establish populations in almost all continents, from tropical to temperate zones, and has developed mechanisms to adapt to adverse conditions, given its physiological plasticity [45]. Our findings are consistent with previous work reporting success in survival and development of immature forms of *Ae. albopictus* and *Ae. aegypti* using dilutions of sea water [23]. However, our approach differs in several aspects. We employed distilled water as the initial medium, devoid of additional minerals, and subsequently introduced varying concentrations of salt. In addition, we not only monitored the survival of immature stages under diverse salinity conditions encompassing a broader spectrum of salt concentrations, but also continued to track them throughout their adult phase. Consequently, we evaluated the potential impact of exposure to varying salinity levels during the immature stages on the adult lifespan.

Our results showed significant differences in the overall survival of the individuals (from L$_1$ to adults), following a decreasing trend in survival as the saline concentrations increased. In larval stages, feeding includes intake of ions under saline concentrations [24]. A plausible explanation for the 24 h-mortality observed for the L_1_ larvae at concentrations above 12 ppt could be the ingestion of high concentrations of NaCl that caused a low assimilation of nutrients due to the increased metabolic demands of osmoregulation [24]. This result is in line with the results reported by Ramasamy et al. [23] that L$_3$ larvae of both *Ae. aegypti* and *Ae. albopictus* were better able to tolerate water salinity than the L_1_. At low concentrations (< 10 ppt), the increase in osmoregulatory-related metabolic costs might not be significant, and thus, survival rates across stages are steady. The Kaplan–Meier curves reinforced the results discussed above, showing that survival in immature stages decreased abruptly as salinity was increased. At low salinity concentrations, however, this pattern showed some exceptions. For instance, at 0.2 and 1 ppt concentrations, survival was significantly higher than with distilled water or at 0.5 ppt. The latter results suggest the activation of adaption mechanisms, or hormetic effects, as a response to insufficient ion concentrations. Hormesis is defined as an adaptive response to stressors which can lead to improved organismal performance at low doses, while high doses may result in detrimental effects that lead to negative consequences [46, 47, 48]. This could explain why individuals with 1 ppt salt concentrations showed higher survival, as in this mild stress condition they could have generated a positive response in their survival, while at 0.2 ppt and 0.5 ppt of salt diluted in distilled water, their survival could have followed the normal pattern or they may have even had a shortage of minerals for their development, as happened with the distilled water condition. We also hypothesize that these mechanisms may produce collateral costs such as emergence of adults of small size, or in a larger proportion of males (that are usually smaller in size than females). However, we have not measured these features, and this is a shortcoming of the current study. Adult survival was apparently not affected by the past environment (i.e., the amount of salinity experienced in different immature stages). Whether other life history traits of adults, such as reproduction, were affected needs to be addressed in a future study. Another notable result was the difference in survival of the larvae growing in distilled water relative to those developing in bottled water. This may be due to the lack of ions and minerals in the distilled water, which could have produced a shortage of the molecules necessary for the correct development and osmosis of the larvae. In Clark et al. [24], individuals of *Ae. aegypti* (freshwater mosquito) and *Ochlerotatus taeniorhynchus* (euryhaline mosquito) showed approximately 80% survival in deionized water, obtaining ions from the food. Both species showed an optimal range of salinity with survival success near 100%, with the optimal concentration for *O. taeniorhynchus* being around 14 g l^−1^, whereas *Ae. aegypti* showed an optimal range of survival from 0 to 3.5 g l^−1^. Other studies have shown that *Ae. aegypti* larvae can survive several days in distilled water, but levels of Na$^+$ and Cl$^-$ in hemolymph drop significantly [49, 50]. Our findings comparing bottled and distilled water conditions are in line with this result, showing an increased risk of death for immature stages developing in distilled water, while posterior survival of adults was not significantly affected.

The study of the life tables reinforced all the previous results. First, life tables for the complete life course of the individuals (L$_1$ to adult death) revealed longer life expectancy (E$_x$) at lower salinity concentrations. The life expectancy of the individuals that grew in distilled water, without the presence of salts or other ions, was reduced than that in bottled water. Indeed, the life expectancy of individuals grown in bottled water was also longer than individuals exposed to higher salt levels throughout their lives. However, the life expectancy of adults was not so different regardless of the different salinity conditions in which they had previously developed. These results are in line with those of Cox proportional hazards models (see Results). While the presence of salt influenced the survival during the immature stages, the life expectancy of the resulting adults was not significantly affected. Hence, the aquatic salinity conditions in which these mosquitoes became adults did not affect their life expectancy as adults.

Our study demonstrates the successful development of *Ae. albopictus* mosquitoes in brackish water under laboratory conditions. Previous research [23] reported the growth of *Ae. albopictus* and *Ae. aegypti* larvae in artificial containers containing salinity concentrations obtained by diluting seawater in tap water, with tolerances observed up to 15 ppt. However, in our study, we observed that mosquitoes did not reach the adult stage when exposed to concentrations higher than 10 ppt. This outcome could be attributed to the absence of other essential ions in our experiment relative to natural seawater, as we combined only distilled water with NaCl to achieve the desired salinity conditions. Furthermore, it is worth noting that the mosquitoes used in our study were laboratory-reared colonies, which may have limited adaptability to changes in the ionic composition of the medium in which immature stages develop. Indeed, as we used the same laboratory populations of *Ae. albopictus*, we could not compare possible different adaptations to salinity concentrations with other populations or other environments. This would allow a more detailed understanding of the possible ecophysiological plasticity of *Ae. albopictus* and should be further studied. Moreover, even though we added a small amount of food to each individual tube with larvae (see Methods), which might have affected the salinity, we were not able to measure the content of salt in the food. Nevertheless, since we maintained a consistent and minimal quantity of food across all salinity concentrations, any potential effect this may have had on the results would have been small and equitable.

Ramasamy et al. [20] documented the potential adaptation of freshwater mosquitoes to brackish water habitats, such as estuaries, coastal marshes, or lagoons, in response to changing water composition caused by global warming and rising sea levels. Although *Ae. albopictus* breeds mainly in artificial and natural containers with retained water and not in open coastal waters, it is important to consider the potential for establishment in new habitats of one of the most invasive species in the world. The authors hypothesized that brackish water development may be an adaptative response to the exclusive application of *Aedes* larvae control measures with insecticides to freshwater habitats and the elimination of such habitats in urban and suburban areas [23]. The surveillance and treatment of freshwater breeding sites may facilitate the potential for development of mosquito larvae in more brackish water, used as a breeding refuge, in the future. Further research is needed to enhance control techniques and expand treatment strategies in tandem with the potential expansion of mosquito habitats from freshwater to brackish and saltwater environments.

## Conclusions

*Aedes albopictus* is one of the most successful invasive mosquitoes, with established populations in both rural and urban environments, covering a range of climates from tropical to temperate. Our findings suggest that although *Ae. albopictus* has been considered as a species that develops strictly in fresh waters, it can develop and survive in brackish waters even without presenting the structures typical of euryhaline species. These new adaptations (yet to be discovered) could facilitate the emergence of new breeding sites and the colonization of new territory in coastal areas by *Ae. albopictus*. Increased knowledge of *Ae. albopictus* physiological adaptations to salinity would allow us to expand the range of control of the species and make more accurate predictions of its dispersal and vector capacity. Comprehensive life tables in salt-related conditions would provide important data for updating habitat suitability modelling and further support the development and application of more efficient control methods integrating a broad range of aquatic ecosystems.

### Supplementary information

**Additional file 1: Table S1.** Chemical analysis of the water used for the bottled water condition. **Table S2.** Parameters, description, notation and formula of the lifetables. **Table S3.** Pairwise comparisons of each group in Kaplan Meier survival analysis of the immature survival (from L1 to adult emergence). **Table S4.** Summary table of the Cox analysis with increasing salinity concentrations and distilled condition as control. **Table S5.** Summary table of the Cox analysis describing the effects of distilled vs. bottled water in the survival.

**Additional file 2:** **Figure S1.** Event-history diagram for each salinity condition. **Figure S2.** Survival as adults by sex; Kaplan–Meier curves estimated for males and females who reached adult stage in each salinity condition.

## Data Availability

The dataset used and the codes to carry out the analysis are available in the following GitHub repository: https://github.com/lblancozgz/Salinity.
