# Supplementary material for: Effect of water salinity on immature performance and lifespan of adult Asian tiger mosquito
Source: Parasit Vectors. 2024 Jan 18;17:24. doi: 10.1186/s13071-023-06069-5 (PMC10797731; doi:10.1186/s13071-023-06069-5)
Supplement: Supplementary file 1 — Additional file 1: Table S1. Chemical analysis of the water used for the bottled water condition. Table S2. Parameters, description, notation and formula of the lifetables. Table S3. Pairwise comparisons of each group in Kaplan Meier survival analysis of the immature survival (from L1 to adult emergence). Table S4. Summary table of the Cox analysis with increasing salinity concentrations and distilled condition as control. Table S5. Summary table of the Cox analysis describing the effects of distilled vs. bottled water in the survival. [file 13071_2023_6069_MOESM1_ESM.pdf]

Table S1. Chemical analysis of the water used for the Bottled water condition.

Composition mg/l-		
Calcium	Ca^{2+}	64
Magnesium	Mg2+	21
Sodium	Na^{+}	8.1
Bicarbonate	HCO_3-	286
Chlorine	Cl-	15
Sulfate	SO_4^2-	6
Nitrate	NO_3-	2
Nitrogen dioxide	NO_{2-}	<0.025

Table S2. Parameters, description, notation and formula of the lifetables

**Parameter or model type**	**Description**	**Notation**	**Formula**
Cohort survival	Fraction alive at age x	l_x	$\frac{N_x}{N_0}$
Age-specific (period) survival	Fraction alive at age x surviving to x+1	p_x	$\frac{l_{x+1}}{l_x}$
Age-specific (period) mortality	Fraction alive at age x dying prior to x+1	q_x	$1-\frac{l_{x+1}}{l_x}$
Death distribution	Fraction of original cohort dying between x and x+1	d_x	$l_x - l_{x+1}$
Expectation of life at age x	Average number of days remaining to individual age x	E_x	$\frac{1}{2}+\frac{l_{x+1}+l_{x+1}+\cdots+l_{\omega}}{l_x}$
Standard deviation	Standard deviation for death distribution (dx)	S_{q_x}	$q_x\sqrt{\frac{1}{d_x}(1-q_x)}$

Table S3. Pairwise comparisons of each group in Kaplan Meier survival analysis of the immature survival (from L1 to adult emergence).

	0.2 ppt	0.5 ppt	1 ppt	2 ppt	5 ppt	10 ppt	Bottled
0.5 ppt	0.221	-	-	-	-	-	-
1 ppt	0.182	0.965	-	-	-	-	-
2 ppt	0.162	0.876	0.747	-	-	-	-
5 ppt	0.084 ·	0.652	0.521	0.905	-	-	-
10 ppt	<0.001***	<0.001***	<0.001***	<0.01**	<0.01**	-	-
Bottled	<0.0001****	<0.0001****	<0.0001****	<0.0001****	<0.0001****	<0.0001****	-
Distilled	0.945	0.200	0.195	0.195	0.173	<0.001***	<0.001***

Salinity groups of 12 ppt, 15 ppt, 20 ppt and 30 ppt excluded from the comparisons as they lived less than 1 day

Table S4. Summary table of the Cox analysis with increasing salinity concentrations and distilled condition as control.

Increasing salinity conditions	Effect	HR	CI95%	p-value
L_1 to adult death	Salinity	1.15	1.13-1.16	<0.0001*
L_1 to adult emergence	Salinity	1.13	1.12-1.14	<0.0001*
Adult lifespan	Salinity	0.99	0.94-1.04	0.661
Adult lifespan	Salinity	0.99	0.94-1.04	0.804
	Sex[b]	1.31	1.04-1.66	<0.05*

Effects denoted with (*) are statistically significant.

[b] Female as reference in the comparison

Table S5. Summary table of the Cox analysis describing the effects of distilled *vs.* bottled water in the survival.

Bottled *vs.* Distilled[a]	Effect	HR	CI95%	p-value
L_1 to adult death	Salinity	1.91	1.42-2.58	<0.0001*
L_1 to adult emergence	Salinity	1.06	0.80-1.41	0.67
Adult lifespan	Salinity	1.29	0.87-1.89	0.19
Adult lifespan	Salinity	1.25	0.84-1.85	0.25
	Sex[b]	0.82	0.56-1.21	0.33

Effects denoted with (*) are statistically significant.

[a] Bottled condition as reference in the comparison.

[b] Female as reference in the comparison
