# Supplementary material for: Effect of water salinity on immature performance and lifespan of adult Asian tiger mosquito
Source: Parasit Vectors. 2024 Jan 18;17:24. doi: 10.1186/s13071-023-06069-5 (PMC10797731; doi:10.1186/s13071-023-06069-5)
Supplement: Supplementary file 2 — Additional file 2: Figure S1. Event-history diagram for each salinity condition. Figure S2. Survival as adults by sex; Kaplan–Meier curves estimated for males and females who reached adult stage in each salinity condition. [file 13071_2023_6069_MOESM2_ESM.pdf]

**Figure S1.** Event-history diagram for each salinity condition.

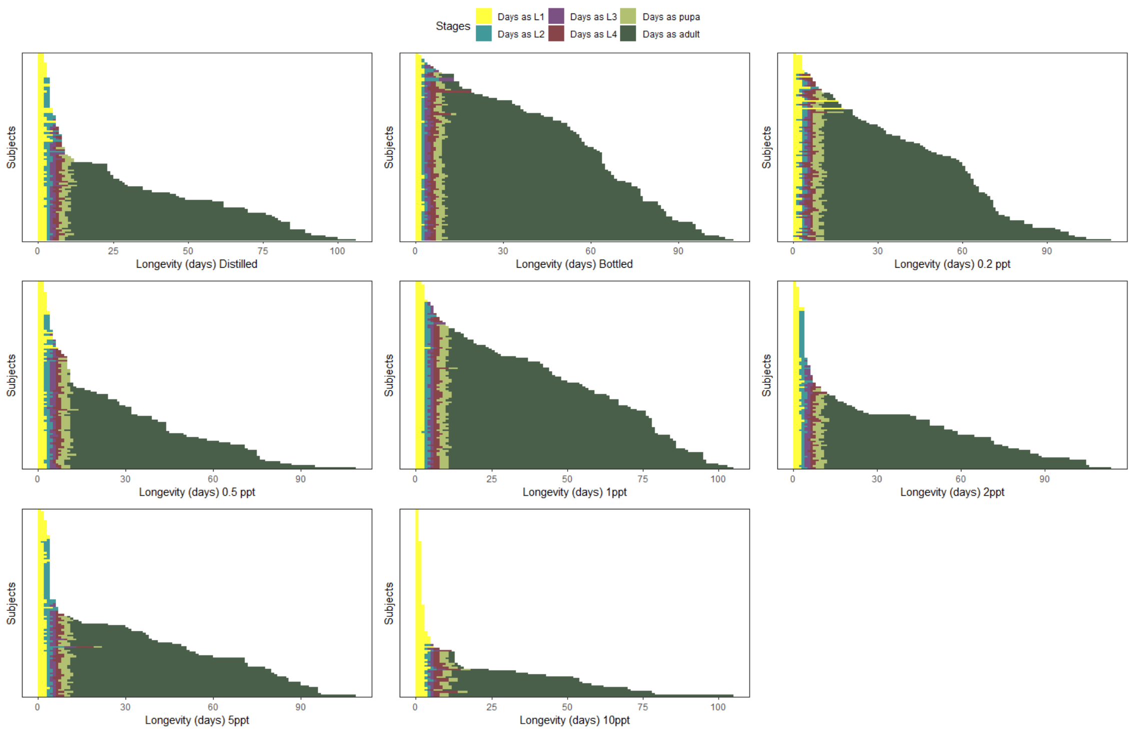


**Figure S2. Survival as adults by sex;** Kaplan-Meier curves estimated for males and females who reached adult stage in each salinity condition.

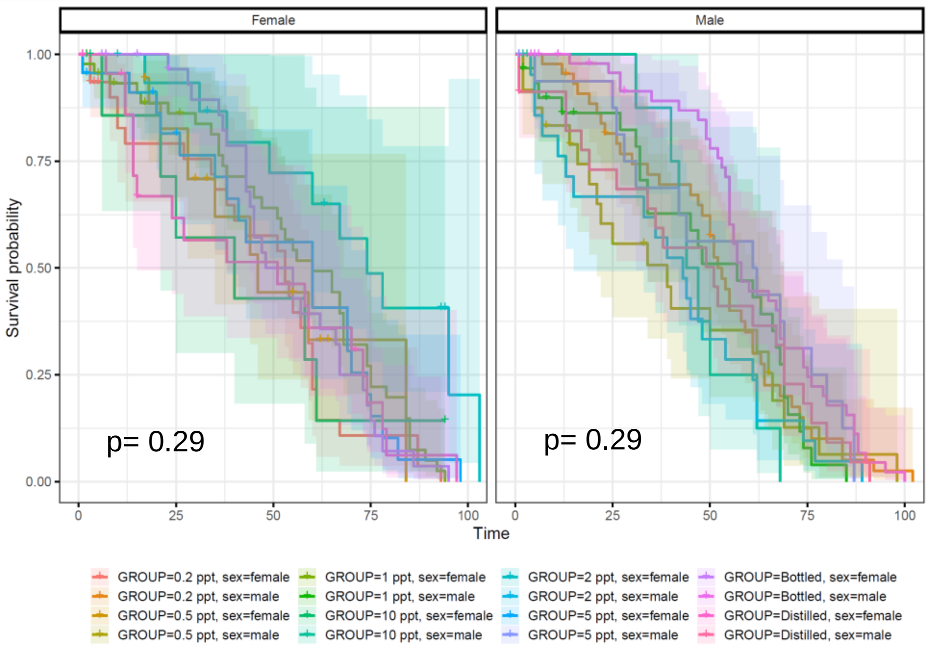


*p-value obtained from the comparison of the survival curves using the log-rank test.
